# *In vivo* Anti-inflammatory Activity of Lipidated Peptidomimetics Pam-(Lys-βNspe)_6_-NH_2_ and Lau-(Lys-βNspe)_6_-NH_2_ Against PMA-Induced Acute Inflammation

**DOI:** 10.3389/fimmu.2020.02102

**Published:** 2020-08-28

**Authors:** Bing C. Wu, Sarah L. Skovbakke, Hamid Masoudi, Robert E. W. Hancock, Henrik Franzyk

**Affiliations:** ^1^Center for Microbial Diseases and Immunity Research, Department of Microbiology and Immunology, The University of British Columbia, Vancouver, BC, Canada; ^2^Biotherapeutic Glycoengineering and Immunology, Department of Biotechnology and Biomedicine, Technical University of Denmark, Kgs Lyngby, Denmark; ^3^Faculty of Medicine, Department of Pathology and Laboratory Medicine, St. Paul’s Hospital, Vancouver, BC, Canada; ^4^Faculty of Health and Medical Sciences, Department of Drug Design and Pharmacology, University of Copenhagen, Copenhagen, Denmark

**Keywords:** peptidomimetics, anti-inflammatory, phorbol 12-myristate 13-acetate, sterile inflammation, formyl peptide receptors, edema, neutrophils, reactive oxygen and nitrogen species

## Abstract

Host Defense Peptides (HDPs) are key components of innate immunity that exert antimicrobial, antibiofilm, and immunomodulatory activities in all higher organisms. Synthetic peptidomimetic analogs were designed to retain the desirable pharmacological properties of HDPs while having improved stability toward enzymatic degradation, providing enhanced potential for therapeutic applications. Lipidated peptide/β-peptoid hybrids [e.g., Pam-(Lys-βNspe)_6_-NH_2_ (PM1) and Lau-(Lys-βNspe)_6_-NH_2_ (PM2)] are proteolytically stable HDP mimetics displaying anti-inflammatory activity and formyl peptide receptor 2 antagonism in human and mouse immune cells *in vitro*. Here PM1 and PM2 were investigated for their *in vivo* anti-inflammatory activity in a phorbol 12-myristate 13-acetate (PMA)-induced acute mouse ear inflammation model. Topical administration of PM1 or PM2 led to attenuated PMA-induced ear edema, reduced local production of the pro-inflammatory chemokines MCP-1 and CXCL-1 as well as the cytokine IL-6. In addition, diminished neutrophil infiltration into PMA-inflamed ear tissue and suppressed local release of reactive oxygen and nitrogen species were observed upon treatment. The obtained results show that these two peptidomimetics exhibit anti-inflammatory effects comparable to that of the non-steroidal anti-inflammatory drug indomethacin, and hence possess a potential for treatment of inflammatory skin conditions.

## Introduction

Inflammation is a defense mechanism of innate immunity that involves complex and well-coordinated networks of cells and signaling molecules (1–3). Thus, major functions of inflammatory processes comprise elimination of pathogens and damaged cells, while the appropriate resolution of inflammation and restoration of homeostasis are crucial to avoid inflammatory disorders (4–7). Inflammation induced by stimulants of non-microbial origin, such as irritants, or so-called damage-associated molecular patterns (e.g., mitochondria-derived formylated peptides) released from damaged cells and tissues during trauma, is referred to as sterile inflammation (8, 9). Excessive sterile inflammation drives non-communicable chronic diseases (e.g., cardiovascular diseases, asthma, rheumatoid arthritis, and chronic obstructive pulmonary disease) that have become leading concerns in public health (10–12). Phorbol 12-myristate 13-acetate (PMA) is a pharmacological activator of protein kinase C, which is a central signaling molecule activated downstream of many inflammatory receptors. Therefore, PMA is often used as a potent inducer of sterile inflammation in screening for the relative activity of potential anti-inflammatory drugs (13–17). Single-dose topical application of PMA to the tissues of the mouse ear induces an acute inflammation characterized by ear swelling, local and systemic secretion of chemokines and cytokines (e.g., MCP-1, CXCL-1, and IL-6) as well as upregulation of cascades involving IFN-γ, TNF, and IL-1 (18). Increased expression of these cytokines has been linked to the pathogenesis of many inflammatory skin disorders, e.g., TNF, IFN-γ, and IL-6 are highly expressed in psoriatic lesions, while IFN-γ and TNF are associated with the chronic phase of atopic dermatitis (19).

Neutrophils constitute the most abundant circulating immune cells that are rapidly recruited to sites of inflammation (e.g., induced via PMA stimulation), where they are major contributors to the local production and release of reactive oxygen and nitrogen species (i.e., ROS/RNS) e.g., as found in ear tissue models. Formyl peptide receptors (FPRs) belong to the class of G-protein-coupled receptors with seven transmembrane domains (20). In neutrophils FPRs are involved in both initiation of inflammatory responses (e.g., assembly and activation of NADPH-oxidase leading to ROS production) and resolution of inflammation, which make FPRs ideal targets for therapeutic intervention (21–26). The subtype FPR1 recognizes the prototypical bacteria- and damage-associated N-formylated peptide agonists with subsequent induction of pro-inflammatory responses, whereas FPR2 recognizes a diverse range of structurally distinct ligands (including lipids, N-formylated, and non-formylated peptides as well as small molecules), and it is involved in both pro-inflammatory and pro-resolving processes (8, 20, 23).

**FIGURE 1:**
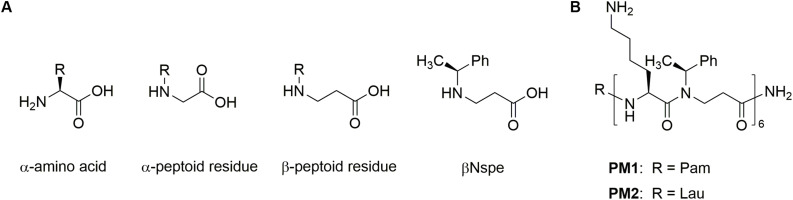
Chemical structures of amino acid peptoid residues **(A)** and peptidomimetics **(B)**.

Host defense peptides (HDPs), also known as antimicrobial peptides, are naturally occurring cationic molecules present in all higher living organisms, where they exert immunomodulatory effects, antibiofilm activity, and broad-spectrum antimicrobial action against pathogens (27, 28). Under physiological conditions, HDPs preferentially modulate innate immune responses by affecting immune cell differentiation, activation, and trafficking, thereby linking innate and adaptive immunity. Many of these immunomodulatory functions of HDPs are mediated through FPRs, resulting in attenuation of sterile and pathogen-induced inflammation as well as promotion of wound healing (22, 29–31). Hence, continuous efforts are devoted to development of HDPs and synthetic mimetics into beneficial therapies (32–34). Since HDPs are inherently susceptible to proteolytic degradation, different approaches for conferring increased stability have been explored: e.g., incorporation of non-natural amino acids, L- to D-amino acid substitution, cyclization, modification of the termini, and formulation with drug delivery systems (35). Peptidomimetics comprise peptide-like molecules with altered backbones that retain side chains similar to those of natural peptides (36). Peptoid oligomers and hybrids with a high content of α- or β-peptoid residues have been found to possess proteolytic stability (34, 37–39). Examples include the immunomodulatory compounds Pam-(Lys-βNspe)_6_-NH_2_ (PM1) and Lau-(Lys-βNspe)_6_-NH_2_ (PM2) that are lipidated peptidomimetics consisting of alternating α-amino acids and β-peptoid residues (see chemical structures in Figure 1) (40–42).

Initially, PM1 was identified from a library of α-peptide/β-peptoid oligomers due to its ability to attenuate, in primary human leukocytes, the production of pro-inflammatory cytokines in response to stimulation with bacterial membrane components including lipopolysaccharide and lipoteichoic acid at concentrations of 60 nM and 0.85 μM, respectively (40) (see Table 1). *In vitro* cellular assays on primary human neutrophils demonstrated that PM1 (at 50 nM) inhibits the release of ROS, neutrophil degranulation, and increases in cytosolic Ca^2+^ concentration upon stimulation with the FPR2-selective peptide agonist WKYMWM (41). Interestingly, the analog PM2, with a four-carbon shorter lipid tail, possesses similar anti-inflammatory properties, albeit at 2- to 4-fold higher concentrations than PM1 (41). Importantly, PM2 proved to be a subtype-selective antagonist of the orthologous mouse receptor, Fpr2, while PM1 antagonized both Fpr1 and Fpr2 signaling (42). Thus, PM2 constitutes the first FPR2 antagonist displaying cross-species selectivity and potency, and thus can be considered to be a convenient tool for elucidating the specific regulatory roles of FPR2 via mouse models of infection and inflammation (see Table 1).

**TABLE 1:** Overview of *in vitro* immunomodulatory activities reported for peptidomimetics PM1 and PM2 (stated as IC_50_ values).

Characteristic	PM1 (μM)	PM2 (μM)	References
Lipopolysaccharide neutralization	0.06 (0.04–0.08)	0.13 (0.08–0.21)	(40)
Lipoteichoic acid neutralization	0.85 (0.5–1.43)	1.84 (1.20–2.82)	(40)
Leukocyte viability	24 (19–30)	27 (18–40)	(40)
HepG2 viability	28 (23–37)	24 (14–42)	(40)
Human FPR2 inhibition	0.05 (0.04–0.07)	0.18 (0.14–0.24)	(41)
Mouse Fpr2 inhibition	+++	0.40 (0.16–0.97)	(42)
Mouse Fpr1 inhibition	++	–	(42)

*+++ = very potent; ++ = potent; and – = inactive.*

In the current first *in vivo* study of these peptidomimetics, we explored the anti-inflammatory effects of PM1 and PM2 by using the PMA-induced mouse ear inflammation model. It was found that both peptidomimetics exhibited potent *in vivo* activity as suppressors of sterile skin inflammation by attenuating PMA-induced ear edema, reducing cytokine and ROS/RNS release, and decreasing neutrophil infiltration in the PMA-inflamed ear tissue to a degree comparable to that of the non-steroidal anti-inflammatory drug (NSAID) indomethacin.

## Results

### Peptidomimetics PM1 and PM2 Dampen PMA-Induced Ear Edema

**FIGURE 2:**
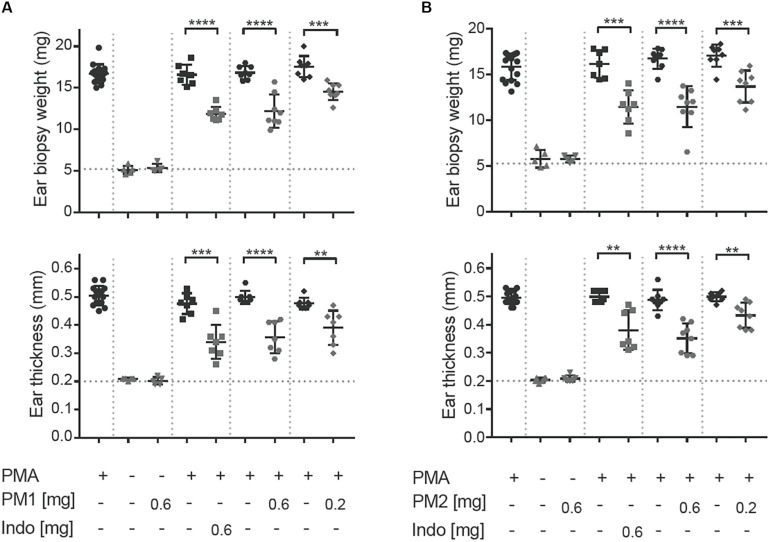
Peptidomimetics PM1 and PM2 reduced PMA-induced ear edema. PMA (20 μL of 125 μg/mL solution) was applied topically onto CD-1 female mice ears. Indomethacin (Indo) or peptidomimetic Pam-(Lys-βNspe)_6_-NH_2_ (PM1, **A**) or Lau-(Lys-βNspe)_6_-NH_2_ (PM2, **B**) at 0.6 or 0.2 mg/ear was given topically to one ear of each mouse after PMA being absorbed. The contralateral ear was given 20 μL of vehicle. Mice were euthanized 6 h post-treatment and increases in ear biopsy weight and ear thickness were measured. Each condition was repeated with a total of five to eight mice in four independent experiments. Error bars indicate Mean ± SD. Statistics: **p ≤ 0.01, ***p ≤ 0.001, and ****p ≤ 0.0001, Student’s unpaired *t*-test.

To induce acute ear inflammation, we applied 20 μL of a 125 μg/mL solution of PMA topically to both ears of CD-1 mice. *In vivo* anti-inflammatory activity of the peptidomimetics was tested by treating one of the PMA-inflamed ears with peptidomimetic PM1 or PM2, while the contralateral ear was given the solvent as a control. The NSAID indomethacin was used as anti-inflammatory positive control in the present study. The ear tissue challenged with PMA started to show signs of inflammation, including swelling and redness about 2 h post-PMA application. These symptoms of inflammation were postponed 3–4 h in ears treated with PM1 and PM2. Consistent with previous findings (18), we observed a ∼3-fold increase in ear biopsy weight and in ear thickness as compared to the vehicle control 6 h post-PMA application in the absence of anti-inflammatory treatment (Figures 2A,B). At the dosages of 0.2 mg/ear and 0.6 mg/ear both peptidomimetics PM1 and PM2 significantly suppressed PMA-induced increases in ear biopsy weight and ear thickness. The anti-inflammatory activity was comparable for both peptidomimetics when applying 0.6 mg/ear and with a matching dose of indomethacin. Application of peptidomimetics alone did not trigger any indications of inflammation. These results infer that HDP mimics PM1 and PM2 both were capable of effectively reducing PMA-induced ear edema.

### PM1 and PM2 Reduce Pro-inflammatory Cytokine and Chemokine Levels in PMA-Inflamed Ear Tissue

**FIGURE 3:**
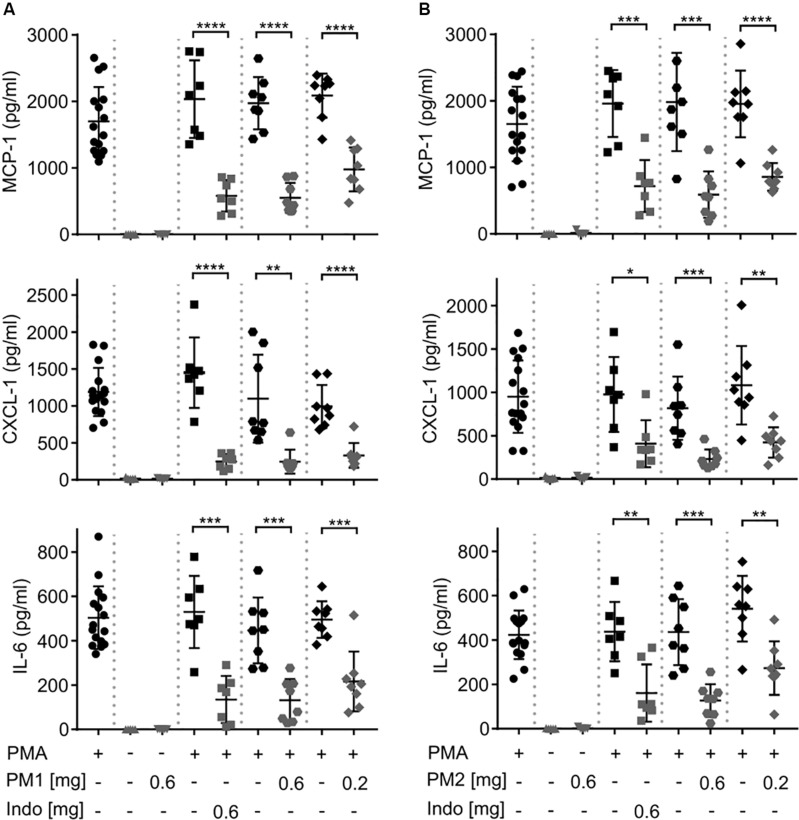
Peptidomimetics PM1 and PM2 attenuated the production of proinflammatory chemokines MCP-1 and CXCL-1 and cytokine IL-6 in PMA-inflamed ear tissue. CD-1 mice were treated as described in Figure 2. Mice ear biopsies (5 mm in diameter) were harvested, homogenized and centrifuged to collect supernatants for determining MCP-1, CXCL-1, and IL-6 levels 6 h post PM1 **(A)** or PM2 **(B)** treatment by ELISA. Each condition was repeated with a total of five to eight mice in four independent experiments. Error bars indicate Mean ± SD. Statistics: *p ≤ 0.05, **p ≤ 0.01, ***p ≤ 0.001, ****p ≤ 0.0001, and Student’s unpaired *t*-test.

To evaluate the local inflammatory processes, we performed ELISA analysis on the ear tissue biopsy 6 h after PMA stimulation (Figure 3). Consistent with a previous study (18), PMA challenge induced significant levels of the chemokines MCP-1/CCL-2, and Gro-α/CXCL-1, as well as the pro-inflammatory cytokine IL-6 in the ear tissue. Topical PM1 treatment, at both tested dosages, significantly decreased MCP-1, CXCL-1, and IL-6 concentrations when compared to those in ears challenged with PMA alone. A similar trend was observed for PM2-treated ear tissue with an approximately 3-fold reduction in MCP-1, CXCL-1, and IL-6 levels in ears treated with 0.6 mg/ear of PM2, and a ∼2-fold reduction for ears that received 0.2 mg/ear of PM2. Both peptidomimetics, at 0.6 mg/ear, suppressed cytokine production to an extent equivalent to that of the positive NSAID control indomethacin. Notably, no induction of MCP-1, CXCL-1, or IL-6 was found in ears treated with PM1 or PM2 alone.

### PM1 and PM2 Only Have Minor Effects on Serum Chemokine and Cytokine Levels

**FIGURE 4:**
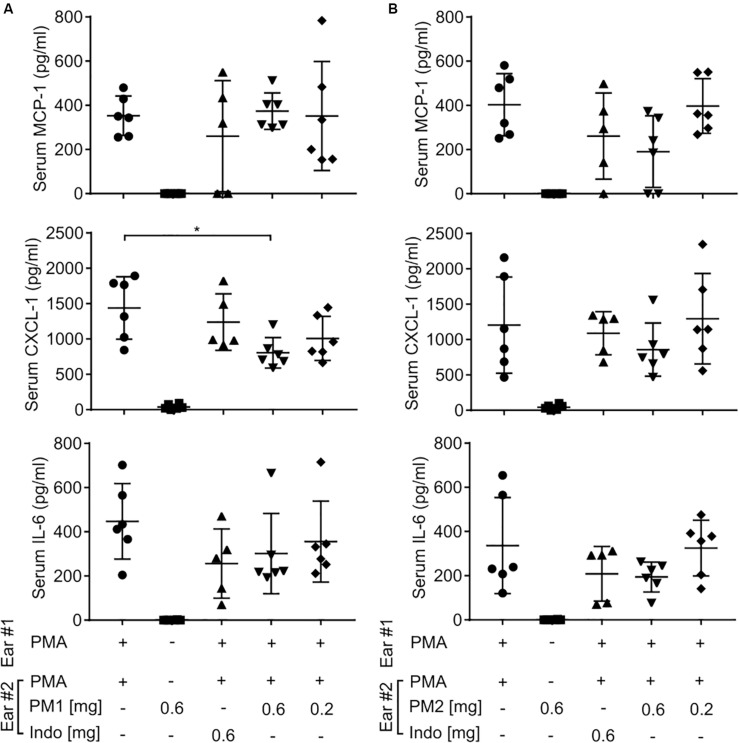
Peptidomimetics PM1 and PM2 only had minor effects on serum chemokine and cytokine levels. CD-1 mice were treated as described in Figure 2. At 6 h post-treatment, serum samples were collected from mice treated with PM1 **(A)** or PM2 **(B)**, and the levels of MCP-1, CXCL-1, and IL-6 were quantified by ELISA. Five to six biological replicates in four independent experiments were included per treatment group. Error bars indicate Mean ± SD. Statistics: *p ≤ 0.05 and Student’s unpaired *t*-test.

To further study whether topical treatment with peptidomimetics PM1 and PM2 exerted a systemic immunomodulatory effect beyond the ear tissue, we harvested mouse blood by cardiac puncture, and then centrifuged the blood to collect serum. Similar to the ear tissue, we measured the content of MCP-1, CXCL-1, and IL-6 in the mouse serum (Figure 4). However, most of these cytokine levels were not affected by the peptidomimetics or indomethacin, with the exception of PM1 giving rise to a decreased serum CXCL-1 concentration upon topical application of 0.6 mg/ear. These results indicate that the anti-inflammatory effects of both peptidomimetics and indomethacin were largely local within the ear tissue.

### PM1 and PM2 Attenuate Neutrophil Recruitment Into PMA-Inflamed Ear Tissue

**FIGURE 5:**
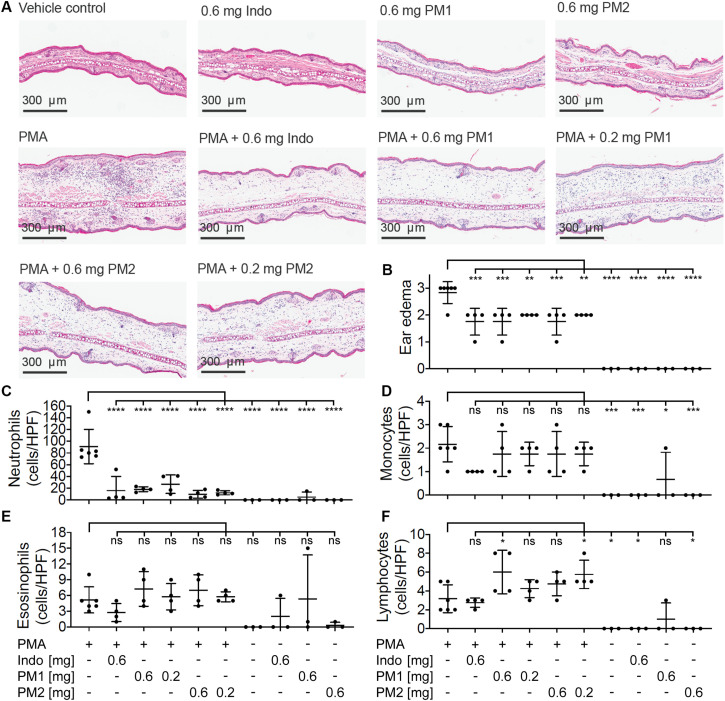
Peptidomimetics PM1 and PM2 suppressed neutrophil infiltration in to the ear tissue. **(A)** A representative image of H&E stained ear tissue biopsies from a total of three to six biological replicates (three independent experiments) per treatment group is shown. **(B)** Ear edema pathology scores (0: no edema, 1: mild, 2: moderate, and 3: severe) were assigned. The number of each type of immune cells present in the stained specimen was determined (cells/HPF): **(C)** neutrophils, **(D)** monocytes, **(E)** eosinophils, and **(F)** lymphocytes. Error bars indicate Mean ± SD. Statistics: *p ≤ 0.05, **p ≤ 0.01, ***p ≤ 0.001, ****p ≤ 0.0001, one-way ANOVA, and Dunnett’s multiple comparisons test.

To evaluate the histologic alterations and inflammatory cell distribution in response to PMA challenge and the treatment with peptidomimetics, hematoxylin and eosin (H&E) staining was performed on tissue biopsies (Figure 5A). In comparison to the sample from the vehicle control tissue, the cross section of ear tissue challenged with PMA exhibited a substantial expansion in the dermal thickness due to increased interstitial fluid. Upon treatment with PM1 or PM2, we observed a prominent decrease in the inflammatory immune cell density and a modest reduction of the dermal thickness. Ear edema scoring revealed that PMA-stimulated ear tissue had moderate to severe edema. Comparable to indomethacin, PM1 or PM2 treatment significantly decreased ear edema scores, reducing the symptoms to moderate on average (Figure 5B). Topical PMA challenge also triggered a marked, predominantly neutrophilic inflammatory infiltrate in the ear tissue (Figure 5C). We also observed significant, but less pronounced, increases in the number of monocytes (Figure 5D) and lymphocytes (Figure 5F), and a minor elevation in eosinophil density (Figure 5E). Treatment with PM1 or PM2 both effectively dampened neutrophil infiltration. In particular, PM2 decreased neutrophil count from an average of 91 cells/High-power field (HPF) to about 10 cells/HPF and 12 cells/HPF for ears treated with 0.6 mg and 0.2 mg PM2, respectively (Figure 5C). Compared to PMA-inflamed ears, PM1 and PM2 treatment did not affect the number of monocytes and eosinophils, but increased the lymphocyte count slightly. These results show that topical treatment with these peptidomimetics can reduce PMA-induced ear edema by preventing excessive influx of neutrophils.

### PM1 and PM2 Reduce the Release of ROS/RNS From PMA-Challenged Ear Tissue

**FIGURE 6:**
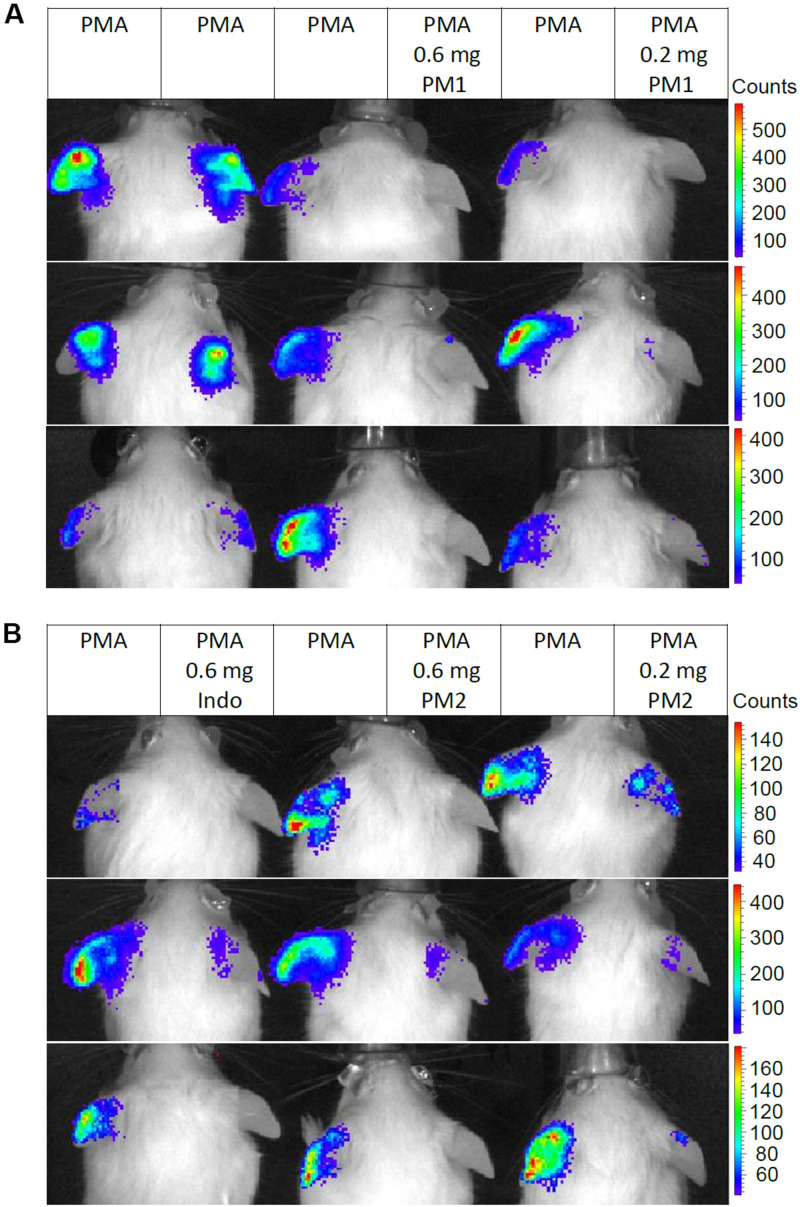
Peptidomimetics PM1 and PM2 attenuated release of ROS/RNS from PMA-challenged ear tissue. Ears of CD-1 mice were stimulated with PMA and the right ear of each mouse was treated with vehicle control (50% acetone), 0.6 mg PM1 or 0.2 mg PM1, respectively, **(A)** or 0.6 mg indomethacin, 0.6 mg PM2 or 0.2 mg PM2, respectively, **(B)**. At 5.5 h post-treatment, CD-1 mice were injected subcutaneously with 25 mg/kg L-012 luminescent probe. ROS/RNS levels in ear tissue were visualized by imaging mice with an *in vivo* imaging system 20–30 min post-probe injection under 2% isoflurane anesthesia. Three biological replicates from three independent experiments were included per treatment group.

We monitored the levels of ROS/RNS, since neutrophil degranulation and the production of ROS/RNS are closely associated with acute inflammatory processes, and an excessive production of these may contribute considerably to the severity of acute inflammation (43, 44). Thus, we injected mice subcutaneously in the back with the luminescent probe L-012 that has high sensitivity toward ROS/RNS and demonstrates enhanced luminescence when binding to these species (45). Subsequently, the mice were subjected to analysis via an *in vivo* imaging system 6 h post-treatment. Figure 6 shows *in vivo* imaging results from three independent experiments. Topical PMA stimulation induced strong ROS/RNS release with some variations among individuals (Figure 6A). Treatment with PM1 at both 0.6 and 0.2 mg/ear almost completely inhibited ROS/RNS production in the ear tissue (Figure 6A), whereas PM2 had a slightly less potent inhibitory effect, being somewhat more effective at the higher dosage (Figure 6B). Again the inhibitory effect on ROS/RNS release was comparable for both peptidomimetics and the positive anti-inflammatory control indomethacin.

## Discussion

Peptidomimetics PM1 and PM2 belong to the class of α-peptide/β-peptoid hybrids with improved proteolytic stability and bioavailability as compared to that of natural HDPs, while retaining beneficial *in vitro* anti-inflammatory properties, including suppression of neutrophil activation and attenuation of pro-inflammatory cytokine production in response to stimulation with bacterial membrane components (40–42, 46). In the present study, we examined the potential of PM1 and PM2 as modulators of PMA-induced sterile inflammation *in vivo*. Consistent with previous studies, topical PMA challenge induced ear tissue swelling, redness, pro-inflammatory cytokine and chemokine production locally in the ear tissue and in the serum within 6 h from PMA application (18, 47, 48). Treatment with PM1 or PM2 effectively reduced PMA-induced ear inflammation, as indicated by their ability to suppress production of MCP-1, CXCL-1, and IL-6 within the ear tissue to an equivalent extent as the NSAID indomethacin positive control (Figure 3). Critically, indomethacin acts via a completely different mechanism, i.e., through potent, non-selective inhibition of the enzyme cyclooxygenase, thereby limiting the production of prostaglandins. Importantly, we observed similar cytokine levels in the PMA-treated negative control ears when comparing mice given PMA only on both ears with mice that had a peptidomimetic applied to one ear, indicating that topical treatment with a peptidomimetic on one ear did not affect the cytokine levels in the contralateral ear. In addition, both peptidomimetics and indomethacin had only minor effects on the serum cytokine levels (Figure 4). Therefore, the anti-inflammatory effect of these peptidomimetics appears to be localized to the ear tissue when applied topically.

Peptidomimetics PM1 and PM2 also had moderate suppressive effects on ear tissue edema as seen by reduced biopsy weight and thickness of the PMA-inflamed ear tissue (Figure 2), which was confirmed by the reduced ear edema score assessed from the histologic sections (Figure 5B). In addition to suppression of dermal expansion, H&E staining showed that the majority of inflammatory cells being recruited to the PMA-inflamed ear tissue were neutrophils, and that treatment with PM1 or PM2 resulted in a prominent reduction in the neutrophil count (Figure 5C), which led to a major decrease in the amount of ROS/RNS accumulated in the ear tissue (Figure 6). In previous studies it was found that pre-incubation of neutrophils with PM1 or PM2 inhibits FPR2-induced ROS production, but not PMA-stimulated ROS secretion from neutrophils *in vitro* (41, 42). A different outcome in the *in vivo* model was expected, since neutrophils are not abundant in healthy skin. Thus, in our *in vivo* ear inflammation model, it did not appear likely that a high number of neutrophils would become directly activated by PMA, which is a receptor-independent protein kinase C activator that promotes release of arachidonic acid from membrane phospholipids. However, products of arachidonic acid metabolism, such as prostaglandins and leukotrienes, increase vascular permeability and evoke infiltration of inflammatory cells, especially neutrophils (49, 50), consistent with the observations presented here. Previous RNA-Seq transcriptomic analysis showed that topical PMA challenge in mouse ears activates cytokine signaling, especially IFN-γ, TNF, and IL-1 as well as chemokine signaling via the class A/1 rhodopsin-like receptor family, and via Toll-like receptor (TLR) signaling pathways (18). Our results thus indicate that PM1 and PM2 likely interfere with these early processes locally, leading to suppression of neutrophil recruitment and activation.

One of the main anti-inflammatory mechanisms of PM1 and PM2 *in vitro* is mediated through inhibition of FPR2/Fpr2 in human and mouse neutrophils (41, 42). Besides neutrophils, FPR2 is also expressed by a variety of immune cells (e.g., monocytes/macrophages, natural killer cells, dendritic cells, and T cells) and non-immune cells (e.g., keratinocytes, intestinal epithelial cells, endothelial cells, and synovial fibroblasts), and they participate in infection responses, pathogenesis of inflammation, and in cancer (25). The detailed functions of FPR2 in skin inflammation have not been well-characterized to date. Activation of FPR2 by PSMα peptides leads to cytokine release, neutrophil chemotaxis and activation during *Staphylococcus aureus* skin infections (51–53). In sterile skin wounds, mouse Fpr1 and Fpr2 have been reported to mediate early neutrophil infiltration into the dermis prior to the production of neutrophil-specific chemokines such as CXCL-1 and CXCL-2 through recognition of FPR ligands produced at the site of injury (54). Also, Fpr1 has been shown to mediate neutrophil accumulation at sites of injury-induced sterile inflammation via recognition of mitochondria-derived formylated peptides (55–57). Consistent with these reports, our results showed that treatment with PM1 or PM2 dampened the initiation of sterile skin inflammation, suppressed ear edema, reduced local cytokine levels and attenuated neutrophil infiltration. In particular, PM1, an antagonist of both Fpr1 and Fpr2 (Table 1), had a better inhibitory effect on local ROS/RNS production as compared to PM2, which is Fpr2-selective (Figure 6). It is likely that the topical PMA challenge resulted in the release of FPR ligands from damaged cells such as keratinocytes, endothelial cells, Langerhans cells in the skin as well as neutrophils and monocytes being recruited to the inflammatory site, and that the effects of PM1 and PM2 in our experimental setting were mediated by Fpr antagonism. Nevertheless, PM1 and PM2 also potently inhibit *in vitro* cytokine secretion induced by stimulation with agonists for TLR-2 and TLR-4. This occurred through cell-dependent mechanisms targeting monocytes and neutrophils (40), indicating that PM1 and PM2 might also inhibit cytokine secretion by TLR-expressing skin-resident cells such as macrophages and Langerhans cells. Further studies are needed to elucidate the detailed molecular mechanism(s) and targeted cell types behind the *in vivo* anti-inflammatory effects exerted by PM1 and PM2.

Together, these results demonstrate that PM1 and PM2 possess promising anti-inflammatory properties *in vivo* against PMA-induced ear inflammation. It is worth mentioning that treatment with PM1 or PM2 was well-tolerated by mice, since we did not observe any signs of piloerection, hunching, extensive scratching or decreased activity for any mice given topical treatment (up to 30 mg/mL) of these two peptidomimetics. Future experiments will focus on characterizing the underlying mechanisms of these peptidomimetics by studying the transcriptomic pathways and networks that these HDP mimics interact with during sterile inflammation.

## Materials and Methods

### Peptidomimetics and Reagents

Peptidomimetics PM1 and PM2 were prepared by a solid-phase synthesis methodology involving assembly of dimeric and/or tetrameric building blocks on a Rink amide resin by using PyBOP as a coupling reagent as earlier reported (58, 59). PMA (≥99% TLC), indomethacin (≥99% TLC), protease inhibitor cocktails, phosphatase inhibitor cocktails 2, and 10% neutral-buffered formalin solution were purchased from Sigma-Aldrich (St. Louis, MO, United States). Tissue Extraction Reagent I was obtained from Thermo Fisher Scientific (Waltham, MA, United States).

### Mice

Animal studies (protocol number A16-0169) were approved by the University of British Columbia Animal Care Committee following the ethical guidelines of the Canadian Council on Animal Care. CD-1 female mice (5 weeks old) were purchased from Charles River Laboratories (Wilmington, MA, United States). Experimental and control mice were co-housed and given standard animal care under controlled room temperature (22 ± 2°C), humidity (40-60%) and a 14 h light and 10 h dark cycle (at the Modified Barrier Facility, University of British Columbia) for at least 1 week before experiments. Mice were divided randomly among different treatment groups on the days of the experiments.

### PMA-Induced Mouse Ear Inflammation Model

The mouse model was carried out as previously published (18). In brief, CD-1 female mice (6–7 weeks old) were anesthetized under 2–5% isoflurane for 10–15 min. During this time, mice were given topical PMA treatment (20 μL of a 125 μg/mL PMA solution in acetone) on both ears to induce acute inflammation. PMA was allowed to air-dry and was fully absorbed before peptidomimetic treatment. Peptidomimetic PM1 or PM2 (20 μL of a 30 mg/mL or a 10 mg/mL solution in 50% ethanol), or the positive control indomethacin (20 μL of a 30 mg/mL solution in acetone) was applied topically onto one ear of each mouse within 3 min after PMA being absorbed. The contralateral ear served as an internal negative control and was given 20 μL of the vehicle 50% ethanol, for mice treated with peptidomimetics, or 20 μL of acetone for mice given indomethacin. At 6 h post-treatment, mice were euthanized using isoflurane anesthetic followed by carbon dioxide, and ear thickness was measured using a digital caliper. Ear biopsies (5 mm in diameter) were harvested using a disposable biopsy punch (VWR), weighted with an analytical balance, homogenized in 600 μL of Tissue Extraction Reagent I supplemented with protease inhibitor cocktails and phosphatase inhibitor cocktails 2, and then centrifuged at 13000 rpm for 20 min at 4°C to collect the supernatant. Blood samples were obtained by cardiac puncture in 1.5 mL microcentrifuge tubes without any anticoagulant. The blood tubes were incubated undisturbed at room temperature for 30 min to allow clotting, and then each tube was centrifuged at 2000 rpm for 10 min, followed immediately by supernatant (serum) collection. Ear tissue supernatant and blood serum were stored at –20°C until cytokine quantification by ELISA.

### ELISA

Mouse CXCL-1 (KC) ELISA Kit was purchased from R&D Systems (Minneapolis, MN, United States). Mouse MCP-1 and IL-6 ELISA kits were purchased from eBioscience (San Diego, CA, United States). Ear tissue and serum cytokine levels were determined according to the manufacturer’s instructions from 5–8 mice per treatment group from 4 independent experiments.

### H&E Staining and Histological Analysis

Ear biopsies (5 mm in diameter), collected 6 h post-treatment, were fixed in 10% neutral-buffered formalin solution at room temperature for 36 h, and then transferred to 70% ethanol. H&E staining of ear tissue cross sections from 3–6 biological replicates (3 independent experiments) per treatment group was performed in a blinded manner by Wax-it Histology Services (Vancouver, BC, Canada); in particular, during the evaluation of the H&E slides, the pathologist (HM) was unaware of the treatment performed for each slide. The ear edema scores were assigned (0: no edema, 1: mild, 2: moderate, and 3: severe) based on the degree of increase in dermal interstitial fluid. The number of each immune cell type including neutrophils, monocytes, eosinophils, and lymphocytes per HPF was quantified from each H&E slide.

### *In vivo* Imaging for ROS/RNS

*In vivo* ROS/RNS detection was performed as described previously (18, 60). In brief, mice were injected subcutaneously with 25 mg/kg L-012 luminescence probe (Wako Chemicals) 5.5 h post-PMA challenge. Mice were imaged under 2% isoflurane anesthesia in groups of 3 using the *in vivo* imaging system (Caliper Life Sciences) 20–30 min post-injection. Images were taken by using Living Image version 3.1 (Caliper Life Sciences) from 3 biological replicates (3 independent experiments) per treatment group.

### Statistical Analyses

Statistical significance was determined using GraphPad Prism Version 8.0.2(159). Comparison between two groups was performed using the Student’s unpaired *t*-test. Comparison among multiple groups was performed using the One-way ANOVA, Dunnett’s multiple comparisons test (^∗^*p* ≤ 0.05; ^∗∗^*p* ≤ 0.01; ^∗∗∗^*p* ≤ 0.001; and ^****^p ≤ 0.0001).

## Data Availability Statement

The raw data supporting the conclusions of this article will be made available by the authors, without undue reservation.

## Ethics Statement

The animal study was reviewed and approved by the University of British Columbia Animal Care Committee following the ethical guidelines of the Canadian Council on Animal Care.

## Author Contributions

BW designed and performed the experiments and analyzed the data. HF and SS designed the peptidomimetics and performed *in vitro* investigations, respectively. HM performed pathology scoring and quantification on the H&E staining samples. All authors contributed to the interpretation of the data and writing, reviewing, and editing the manuscript. RH and HF supervised the research and acquired the funding.

## Conflict of Interest

The authors declare that the research was conducted in the absence of any commercial or financial relationships that could be construed as a potential conflict of interest.

## Abbreviations

FPR/Fpr: Formyl peptide receptor (in human/mouse)
HDP: Host defense peptide
H&E: Hematoxylin and eosin
HPF: High-power field
NSAID: non-steroidal anti-inflammatory drug
PMA: Phorbol 12-myristate 13-acetate
PM1: Pam-(Lys- β Nspe)_6_-NH_2_
PM2: Lau-(Lys- β Nspe)_6_-NH_2_
RNS: Reactive nitrogen species
ROS: Reactive oxygen species.
